# An In Vivo Model of Propionic Acid-Rich Diet-Induced Gliosis and Neuro-Inflammation in Mice (FVB/N-Tg(GFAPGFP)14Mes/J): A Potential Link to Autism Spectrum Disorder

**DOI:** 10.3390/ijms25158093

**Published:** 2024-07-25

**Authors:** Piotr P. Lagod, Latifa S. Abdelli, Saleh A. Naser

**Affiliations:** 1 Burnett School of Biomedical Sciences, College of Medicine, University of Central Florida, Orlando, FL 32816, USA; piotr.lagod@ucf.edu; 2 Health Sciences Department, College of Health Professions and Sciences, University of Central Florida, Orlando, FL 32816, USA; latifa.abdelli@ucf.edu

**Keywords:** autism, propionic acid, astrocytes, neuro-inflammation, gliosis, FVB/N-Tg(GFAPGFP)14Mes/J mice

## Abstract

Evidence shows that Autism Spectrum Disorder (ASD) stems from an interplay of genetic and environmental factors, which may include propionic acid (PPA), a microbial byproduct and food preservative. We previously reported that in vitro treatment of neural stem cells with PPA leads to gliosis and neuroinflammation. In this study, mice were exposed ad libitum to a PPA-rich diet for four weeks before mating. The same diet was maintained through pregnancy and administered to the offspring after weaning. The brains of the offspring were studied at 1 and 5 months postpartum. Glial fibrillary acidic protein (astrocytic marker) was significantly increased (1.53 ± 0.56-fold at 1 M and 1.63 ± 0.49-fold at 5 M) in the PPA group brains. Tubulin IIIβ (neuronal marker) was significantly decreased in the 5 M group. *IL-6* and *TNF-α* expression were increased in the brain of the PPA group (IL-6: 2.48 ± 1.25-fold at 5 M; TNF-α: 2.84 ± 1.16-fold at 1 M and 2.64 ± 1.42-fold, at 5 M), while IL-10 was decreased. GPR41 and p-Akt were increased, while PTEN (p-Akt inhibitor) was decreased in the PPA group. The data support the role of a PPA-rich diet in glia over-proliferation and neuro-inflammation mediated by the GPR41 receptor and PTEN/Akt pathway. These findings strongly support our earlier study on the role of PPA in ASD.

## 1. Introduction

Autism Spectrum Disorder (ASD) is a neurodevelopmental condition with a complex etiology that is still only sparsely understood [1]. It is characterized by repetitive and restrictive behavior, deficits in communication and social interactions, and a wide range of possible cognitive impairments [2,3,4,5,6,7]. ASD’s prevalence quadrupled in the last 20 years, and according to the newest statistics, 1:36 children are affected [8]. The only interventions that are currently available are costly and burdensome behavioral therapies aiming at best to diminish symptoms and increase patients’ independence. A staggering rise in prevalence and lack of effective treatment continues to burden patients, their families, and society. This daunting reality underlines the great need to better understand the etiology of ASD and all factors involved, in hopes of providing novel treatment approaches, therapeutics, and preventative measures [4,9,10].

Evidence shows that ASD stems from a complex interplay of genetic and environmental factors, such as toxins, oxidative stress, metabolic abnormalities, and immune dysregulation during the gestational period [11,12,13,14]. In recent years, propionic acid (PPA), a short-chain fatty acid, gained some momentum, as it was found to be elevated in the stool of ASD patients [15,16,17,18]. Both mothers and their children were reported to have significantly elevated levels of gut bacteria that produce a high amount of PPA as a byproduct of carbohydrates and dietary fiber fermentation, especially *Clostridium*, *Bacteroides*, and *Desulfovibrio* [1,19,20,21,22,23,24]. PPA and its salts (potassium propionate, sodium propionate, and calcium propionate) are common food additives used in the food industry for their antifungal properties [25,26].

PPA can cross the blood–brain barrier, where it can modulate cell signaling, lipid and energy metabolism, and the synthesis and release of neurotransmitters [12,27]. However, an excess of PPA in circulation may have deleterious effects. This has been reported in Propionic Acidemia, in which an accumulation of PPA (due to Propionyl CoA Carboxylase dysfunction) leads to symptoms that are associated with ASD, such as movement disorders, seizures, and developmental delays [28,29,30]. Additionally, several studies showed that intracerebral injection of PPA evokes an autism phenotype in rats, which includes behavioral changes, disturbances in the metabolism, over-proliferation of neuroinflammatory macrophages (microglia), and increased levels of inflammatory markers, such as IL-6, TNF-*α*, and Interferon-γ [27,31,32,33,34].

Clinical studies involving the analysis of cerebrospinal fluid and post-mortem brain tissue showed an increase in key pro-inflammatory cytokines [34,35,36,37] and a decrease in anti-inflammatory cytokines in ASD patients versus age-matched controls [38].

Interestingly, a recent study conducted by our group revealed that in vitro exposure of human neural stem cells to PPA radically shifted their differentiation toward glial cells. The data showed significantly more cells expressing glial markers (glial fibrillary acidic protein; GFAP) and significantly fewer cells expressing neuronal markers (Tubulin-IIIβ) when exposed to PPA versus the control group [4]. These data suggest a plausible link between PPA and over-proliferation of glial cells associated with many neurodevelopmental disorders, including ASD [39,40,41,42]. Glial cells are involved in neuronal development, protection, and connectivity; this includes homeostasis maintenance, neurotransmitter clearance, and metabolic support of neurons. In response to the toxin and cellular damage, as reported in traumatic brain injury, astrocytes (a glial cell subtype) release inflammatory cytokines, causing neuro-inflammation. The creation of a pro-inflammatory microenvironment contributes to abnormal neuronal activity and a significant increase in oxidative stress. Additionally, in the state of astrogliosis, astrocytes release GFAP and molecules responsible for the creation of the extracellular matrix, such as chondroitin sulfate proteoglycans, which block the regeneration of axons and lead to the formation of glial scars [4,43,44,45,46]. With such deleterious effects on the adult brain, one can easily speculate on the dramatic consequences that might result from glial cell over-proliferation in the earliest stages of neural patterning. Studies have shown that ASD patients have significantly higher levels of GFAP in their serum versus neurotypical controls. Moreover, brain autopsies revealed elevated levels of GFAP in the parietal, superior frontal, and cerebellar cortices of patients with ASD versus typical counterparts, therefore suggesting an over-proliferation of GFAP-producing cells (glia). However, it remains unclear as to how glia over-proliferation occurs in the ASD brain [47,48,49].

Our previous work proposed a possible pathway by which PPA exerts its effect on hNSCs and shifts their differentiation pattern towards the glial phenotype in vitro [4]. We were able to establish that PPA induced its effect through GPR41 (G protein-coupled receptor 41), also known as FFAR3 (Free Fatty Acid Receptor 3), which is predominantly expressed in glial cells [4,50,51]. Our in vitro data showed PPA binding to its receptor, downregulated PTEN (phosphatase and tensin homolog; an inhibitor of Akt phosphorylation), leading to enhanced Akt phosphorylation and activation of the cell survival pathway in glia to the detriment of neurons. Our previous data corroborated ulterior reports describing that spatiotemporal knockout of PTEN in the mouse model led to ASD-like symptoms, neuroinflammation, and alterations in brain development [52,53]. Based on these data, we aimed to reproduce a similar phenomenon in an in vivo mouse model.

Taking into consideration the following, (1) mothers and their ASD children have altered microbiota with a shift towards species producing PPA [1,16,17,54], (2) PPA can induce glial proliferation and a pro-inflammatory cytokine profile in vitro [4], (3) PPA is heavily used in processed food as an antifungal agent, and (4) intracerebroventricular injection of PPA can cause ASD-like symptoms in rats [27,31,55], we hypothesize that PPA would disrupt neural patterning, contributing to the development of gliosis and neuroinflammation during the earliest stages of neural patterning, mimicking ASD. Additionally, we hypothesize that further subjection of offspring to PPA via the diet will exacerbate ASD-associated inflammation. To test this hypothesis, we exposed mice to PPA in utero via the maternal diet (and post-weaning via PPA-rich chow), and after which we tested the offspring at 1 month (adolescents) and 5 months (adults) post-partum for signs of gliosis, astrocyte over-proliferation, and inflammation.

## 2. Results

### 2.1. Fluorescence from the GFAP-GFP Construct Is Significantly Elevated in the PPA-Rich Diet Group

**Figure 1:**
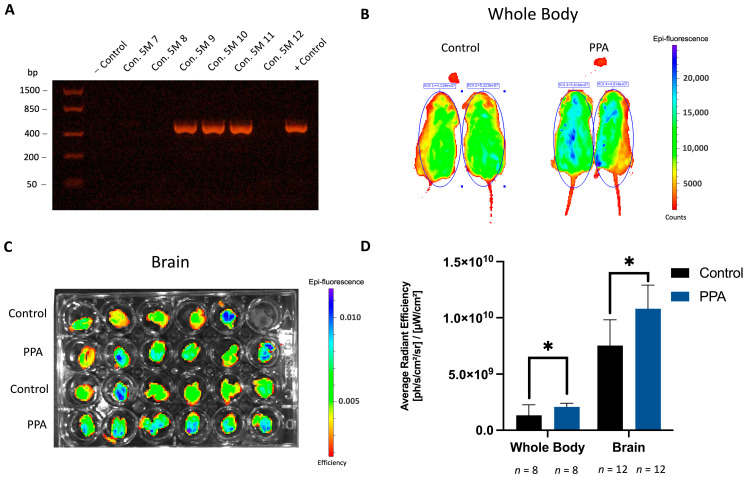
Offspring in the PPA-rich diet groups exhibit a significant increase in fluorescence from the GFAP-GFP construct. Subfigure (**A**) contains a representative image of the touchdown PCR products, indicating the presence of the GFAP-GFP construct. Subfigure (**B**) contains a representative image of whole-body in vivo fluorescence. Subfigure (**C**) represents brain ex vivo fluorescence from the GFAP-GFP construct. Subfigure (**D**) represents the quantified average radiant efficiency [ph/s/cm^2^/sr]/[µW/cm^2^]. The data are represented as mean + SD; *n* = 8 and *n* = 12 for the whole body and ex vivo brain, respectively. The *p*-value < 0.05 is considered significant (*). Abbreviations: PPA (propionic acid), GFAP-GFP (glial fibrillary acidic protein-green fluorescent protein), SD (standard deviation), ph (photons), sr (steradian).

To test if a PPA-rich maternal diet and post-natal exposure to PPA can lead to overproliferation of astrocytes in offspring, mice expressing GFP under the glia-specific promoter (GFAP) were used. To ensure that the GFP construct was present, genotyping was performed on tail snips obtained from all offspring. The amplification product at 498 bp signifies positive results for the construct. Figure 1A shows the representative results where animals designated as Con, 5 M (9, 10, and 11) were positive (inherited and were carrying the construct), while 7, 8, and 12 were negative (did not inherit the construct from their parent).

In vivo whole-body fluorescence and ex vivo brain fluorescence were measured in positive animals from the 5-month (5 M) study. Animals from the 1-month (1 M) study were not subjected to in vivo whole-body fluorescence, due to technical limitations. The average radiant efficiency of the whole body was significantly elevated (Figure 1D, *p* < 0.05, *n* = 8 for both the control and PPA groups), in which the mean for the control was 1.325165 × 10^9^ (±9.385792847 × 10^8^) [ph/s/cm^2^/sr]/[µW/cm^2^] and the mean for the PPA group was 2.08 × 10^9^ (±3.15 × 10^8^) [ph/s/cm^2^/sr]/[µW/cm^2^]. Figure 1B shows the representative animals placed in a ventral position and noses tucked in the Isoflurane delivery system within the imaging unit. Bright blue represents the brighter epi fluorescence detected and measured. To circumvent any fluorescence loss due to bony structures and other tissues found in the whole-body fluorescent imaging, isolated brains were also imaged (Figure 1C). The resulting data further showed that average radiant efficiency was significantly elevated (*p* < 0.05, *n* = 12) in offspring from dams maintained on a PPA-rich diet (and maintained on the same diet until the time of sacrifice at 5 months). In the PPA group, it was equal to 1.08 × 10^10^ (±2.1 × 10^9^) [ph/s/cm^2^/sr]/[µW/cm^2^] against the control 7.54 × 10^9^ (±2.28 × 10^9^) [ph/s/cm^2^/sr]/[µW/cm^2^].

### 2.2. GFAP Is Significantly Elevated in the Brain of Animals in the PPA-Rich Diet Group While Tubulin III β Is Decreased

**Figure 2:**
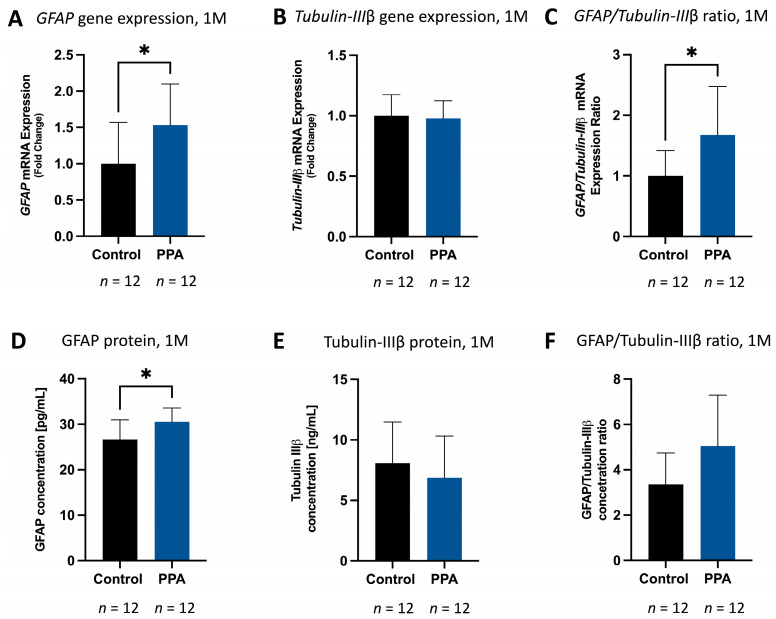
Expression and concentration of GFAP, Tubulin-IIIβ, and GFAP/Tubulin-IIIβ ratio in the brain in the 1 M study. Subfigures (**A**–**C**) represent gene expression calculated using the 2^−ΔΔCT^ method and normalized to *GAPDH*. Subfigures (**D**–**F**) represent protein concentration. The data are represented as mean + SD; *p*-value < 0.05 is considered significant (*). The sample sizes are indicated below each bar graph. Abbreviations: PPA (propionic acid), GFAP (glial fibrillary acidic protein), SD (standard deviation), 1 M (1 month), 5 M (5 months).

**Figure 3:**
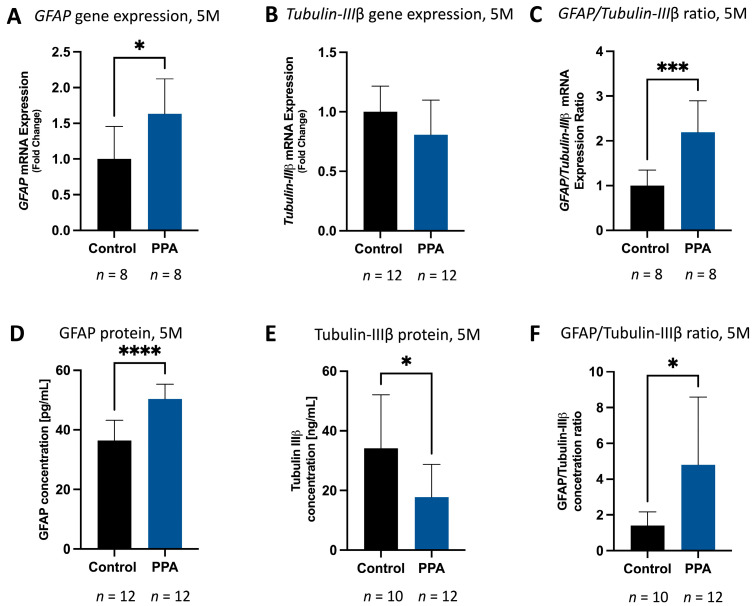
Expression and concentration of GFAP, Tubulin IIIβ, and GFAP/Tubulin IIIβ ratio in the brain in the 5 M study. Subfigures (**A**–**C**) represent gene expression calculated using the 2^−ΔΔCT^ method and normalized to *GAPDH*. Subfigures (**D**–**F**) represent protein concentration. The data are represented as mean + SD; *p*-value < 0.05 (*), *p* < 0.001 (***), and *p* < 0.0001 (****) are considered significant. The sample sizes are indicated below each bar graph. Abbreviations: PPA (propionic acid), GFAP (glial fibrillary acidic protein), SD (standard deviation), 1 M (1 month), 5 M (5 months).

To test how PPA in the maternal diet (1 M) alongside post-natal exposure (5 M) would affect the proliferation of astrocytes and neurons in the brain of offspring, we measured gene expression and protein concentration of astrocytic (GFAP) and neuronal (Tubulin IIIβ) markers in the 1-month-old (adolescent) and 5-month-old (adult) mice. At 1 M, both GFAP gene expression and protein concentration were significantly elevated (*p* < 0.05, *n* = 12 for both the control and PPA groups). Relative *GFAP* expression (measured with the 2^−ΔΔCT^ method and normalized to *GAPDH*) in the PPA group was 1.53 (±0.56)-fold higher versus the control group (Figure 2A). Similarly, GFAP protein levels were significantly elevated (*p* < 0.05, *n* = 12). In the control group, the concentration of GFAP was 26.69 (±4.29) [pg/mL], whereas in the PPA group, it spiked to 30.53 (±3.60) [pg/mL] (Figure 2D). Interestingly, there was no difference in Tubulin IIIβ gene expression and protein levels in the control and PPA groups (Figure 2B and Figure 2E, respectively). However, of note, *GFAP/Tubulin IIIβ* RNA expression was significantly elevated (*p* < 0.05, *n* = 12) in the PPA versus the control group, with an increasing trend in protein levels (Figure 2C and Figure 2F, respectively). To further determine if the elevated expression of the GFAP marker persists to adulthood (5 M) and was exacerbated by subsequent exposure to a PPA-rich diet, the same round of analysis was conducted on the 5 M group. Unsurprisingly, similar results were observed, but with an even more pronounced difference between the control and PPA groups. Specifically, the mean RNA expression of *GFAP* in the PPA group was 1.63 (±0.49)-fold higher (statistically significant, *p* < 0.05, *n* = 8) than the control group (Figure 3A), while protein concentration was 50.36 (±4.99) [pg/mL] in the PPA group versus 36.42 (±6.734) [pg/mL] in the control group (Figure 3D) (*p* < 0.0001, *n* = 12). *Tubulin IIIβ* gene expression showed a diminished trend in the PPA versus the control group (Figure 3B), while Tubulin IIIβ protein concentration was significantly decreased (*p* < 0.05, *n* = 12) from 34.17 (±17.92) [ng/mL] in the control to 17.73 (±11.03) [ng/mL] in the PPA group (Figure 3E). The ratios of *GFAP/Tubulin IIIβ* gene expression (Figure 3C, *p* < 0.001, *n* = 8) and protein concentration (Figure 3F, *p* < 0.05, *n* = 10 in the control and n = 12 in the PPA group) were also significantly elevated in the PPA versus the control group.

### 2.3. A PPA-Rich Diet Correlates with Elevated GPR41 and Phosphorylated Akt and Decreased PTEN Expression and Protein Concentration in Offspring Brains

**Figure 4:**
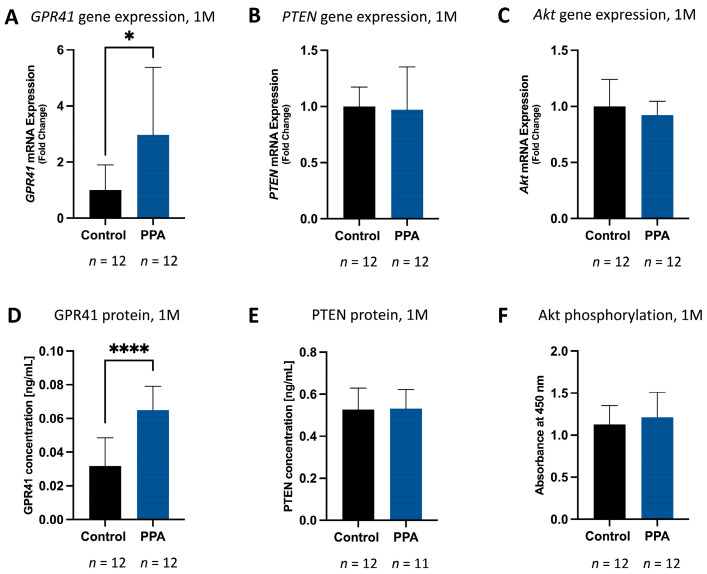
The effect of PPA on GPR41 and PTEN gene expression and protein concentration as well as Akt phosphorylation in the 1 M study. Subfigures (**A**–**C**) represent the expression of *GPR41*, *PTEN*, and *Akt*, respectively, at 1 month of age measured using the 2^−ΔΔCT^ method and normalized to *GAPDH*. Subfigures (**D**,**E**) represent the concentration of GPR41 and PTEN, respectively, in ng/mL measured with an ELISA kit. Subfigure (**F**) shows the relative phosphorylation of Akt reported as absorbance at 450 nm measured with an ELISA kit. All the data within this figure are presented as mean + SD; *p*-value < 0.05 (*) and *p* < 0.0001 (****) are considered significant. The sample sizes are indicated below each bar graph. Abbreviations: PPA (propionic acid), GPR41 (G protein-coupled receptor 41), PTEN (phosphatase and tensin homolog), SD (standard deviation), 1 M (1 month), 5 M (5 months).

**Figure 5:**
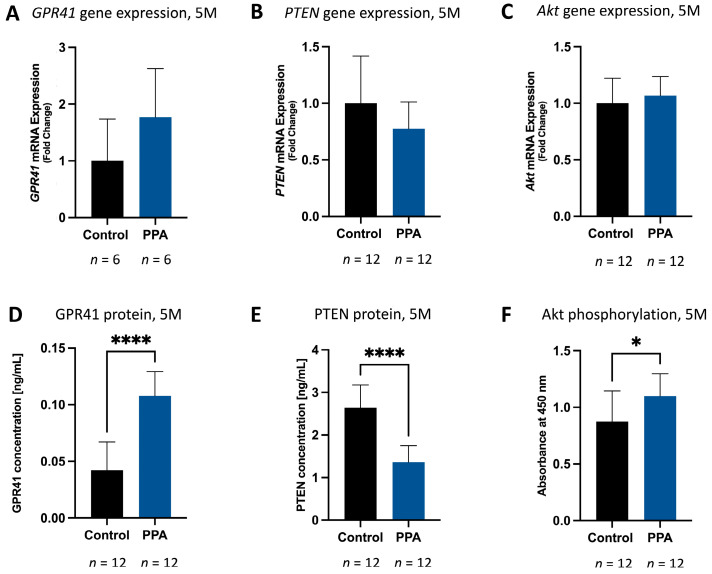
The effect of PPA on GPR41 and PTEN gene expression and protein concentration as well as Akt phosphorylation in the 5 M study. Subfigures (**A**–**C**) represent the expression of *GPR41*, *PTEN*, and *Akt*, respectively, at 5 months of age, normalized to *GAPDH* (utilizing the 2^−ΔΔCT^ method). Subfigure (**D**) represents the concentration of GPR41, and subfigure (**E**) represents the concentration of PTEN, both in ng/mL. Subfigure (**F**) shows the relative phosphorylation of Akt reported as absorbance at 450 nm. The data presented in figures (**D**–**F**) were obtained with the use of ELISA kits. The data are represented as mean + SD, and *p*-value < 0.05 (*) and *p* < 0.0001 (****) are considered significant. The sample sizes are indicated below each bar graph. Abbreviations: PPA (propionic acid), GPR41 (G protein-coupled receptor 41), PTEN (phosphatase and tensin homolog), SD (standard deviation), 1 M (1 month), 5 M (5 months).

In the 1 M study, *GPR41* expression was significantly elevated (*p* < 0.05, *n* = 12 for both the control and PPA groups) in the PPA group compared to the control (2.97 ± 2.40-fold increase, Figure 4A). The corresponding protein concentration (Figure 4D) was also significantly elevated (*p* < 0.0001, *n* = 12), with a mean concentration of 0.032 (±0.018) [ng/mL] in the control group and 0.065 (±0.014) [ng/mL] in the PPA group. In the 5 M study, a similar trend, albeit not significant, was observed, with an increase in *GPR41* RNA expression in the PPA diet group (Figure 5A). Protein concentration was significantly increased (*p* < 0.0001, *n* = 12) in the PPA diet group, with a mean concentration of 0.042 (±0.025) [ng/mL] in the control and 0.108 (±0.021) [ng/mL] in the PPA diet group (Figure 5D). Furthermore, changes in *PTEN* gene expression and protein concentration in the 1 M study were negligible (Figure 4B,E); however, there was an observed decrease in the *PTEN* expression trend (Figure 5B) and a statistically significant (*p* < 0.0001, *n* = 12) decrease in protein concentration in the PPA versus the control group in the 5 M timeline. The mean PTEN concentration was 2.64 (±0.54) [ng/mL] in the control and 1.36 (±0.387) [ng/mL] in the PPA group (Figure 5E).

Of note, although the relative expression of *Akt* was not affected by PPA in either the 1 M or 5 M studies (Figure 4C and Figure 5C, respectively), the relative phosphorylation of Akt was slightly increased in the 1 M study (Figure 4F) and reached significance (*p* < 0.05, *n* = 12) in the PPA versus the control group in the 5 M study (Figure 5F).

### 2.4. A Maternal PPA-Rich Diet and Post-Natal Exposure Increases Expression of Offspring Pro-Inflammatory Cytokines and Decreases Expression of Anti-Inflammatory Cytokines

**Figure 6:**
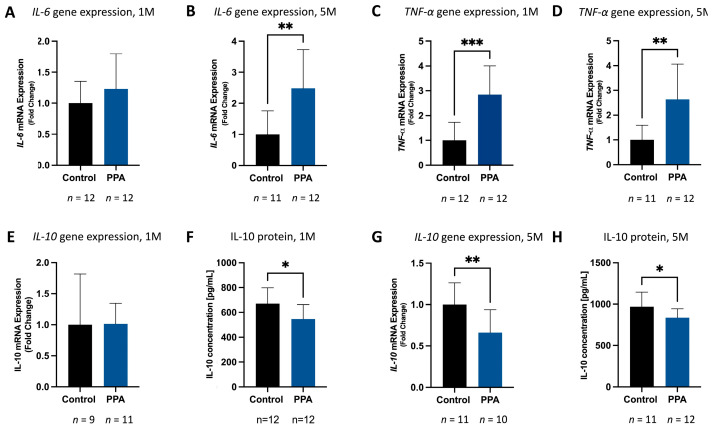
The effect of PPA on the expression of pro- and anti-inflammatory cytokines in the 1 M and 5 M studies. Subfigures (**A**,**B**) represent RNA expression of *IL-6* in the 1 M and 5 M studies, respectively. Subfigures (**C**,**D**) represent RNA expression of *TNF-α* in the 1 M and 5 M studies, respectively. Subfigures (**E**,**G**) represent RNA expression of *IL-10* in the 1 M and 5 M studies, respectively. Subfigures (**F**,**H**) represent protein concentration of IL-10 in the 1 M and 5 M studies, respectively. The expression data for each gene were calculated using the 2^−ΔΔCT^ method and normalized to *GAPDH*, while protein concentration was measured with an ELISA kit. The data are represented as mean + SD; *p*-value < 0.05 (*), *p* < 0.01 (**), and *p* < 0.001 (***) are considered significant. The sample sizes are indicated below each bar graph. Abbreviations: PPA (propionic acid), SD (standard deviation), 1 M (1 month), 5 M (5 months).

To determine the effect of a PPA diet on neuroinflammation, we measured the expression of key pro-inflammatory cytokines in the brain: *TNF-α* and *IL-6*. At 1 M, the expression of pro-inflammatory *IL-6* showed an increasing trend in the PPA versus the control group (1.23 (±0.568)-fold difference, Figure 6A). At 5 M, similar results were obtained, but with a greater fold difference that reached statistical significance (*p* < 0.01, *n* = 11 in the control and *n* = 12 in the PPA group); there was a 2.48 (±1.25)-fold increase in the PPA versus the control group (Figure 6B). *TNF-α* (another key pro-inflammatory cytokine) was also significantly increased in both the 1 M (*p* < 0.001, n = 12) study and 5 M (*p* < 0.01, *n* = 11 in the control and *n* = 12 in the PPA group). At 1 M, a 2.84 (±1.16)-fold increase in the PPA versus the control group was observed (Figure 6C), while at 5 M, a 2.64 (±1.42)-fold increase was observed (Figure 6D). Finally, we also measured gene expression and protein concentration of IL-10 (an anti-inflammatory cytokine). At 1 M, there was no difference in *IL-10* expression between the groups (Figure 6E); however, there was a significant decrease in protein concentration (*p* < 0.05, *n* = 12), with a 639.7 (±75.4) [pg/mL] reading in the control group versus 546.1 (±116.4) [pg/mL] in the PPA diet group (Figure 6F). At 5 M, *IL-10* expression was significantly diminished (*p* < 0.01, *n* = 11 in the control and *n* = 10 in the PPA group) in the PPA diet group (0.66 ± 0.28-fold decrease, Figure 6G). Similarly, IL-10 protein concentration was significantly decreased (*p* < 0.05, *n* = 11 in the control and *n* = 12 in the PPA group) in the PPA group, with readings of 834.4 (±108.8) [pg/mL] versus the control group readings of 969.8 (±176.1) [pg/mL] (Figure 6H).

## 3. Discussion

The currently limited knowledge regarding ASD etiology indicates an intricate interplay between genetic factors and environmental triggers [56,57]. In this current study, we aimed at elucidating the potential role of the maternal diet during the gestational period and the role of a PPA-rich diet administered to offspring post-weaning on neural stem patterning, glial proliferation, and neuroinflammation in offspring. Interestingly, our laboratory previously established that PPA (a food preservative) treatment derails neural stem cell differentiation in vitro, leading to an increase in the ratio of glial cells (80% of cells) [4]. Furthermore, ulterior reports demonstrated an increase in PPA-producing bacteria in stools collected from both mothers and ASD children [1]; this shift includes elevated *Clostridia*, *Bacteriodetes*, and *Desulfovibrio* in ASD. PPA and its salts are also widely used as a preservative in processed food due to their anti-fungal characteristics.

Attempts to reproduce autistic features in rodents using PPA have been reported, where direct intracerebroventricular delivery of PPA in rats resulted in autistic-like behavior and an increased pro-inflammatory profile (IL-6, TNF-α, and interferon-γ). However, it remained unclear if higher doses of PPA in the maternal diet during the gestational period could trigger an autistic phenotype in their progeny (1 M) and/or if it could be subsequently exacerbated by post-weaning exposure to a PPA-rich diet (5 M). In this current study, we hypothesized that a PPA-rich diet would interfere with early neural patterning, causing glia over-proliferation and neuro-inflammation in mice progeny.

Gliosis has been reported in many neurodegenerative and neurodevelopmental diseases, including ASD. A state of gliosis is established by increased glial proliferation, activation, and elevated GFAP (a classical astrocytic marker) release in the brain [47,48]. In our study, we observed a significant increase in astrocyte proliferation in the group subjected to a PPA diet, as evidenced by a significant increase in the average radiant efficiency in whole-body and brain fluorescence readings at 5 M. The GFAP-GFP construct is extremely useful in this setting, as increased GFP fluorescence directly correlates with increased GFAP expression. Noteworthily, since bony structures interfere with fluorescence detection in the IVIS system, we opted to circumvent this limitation by measuring fluorescence on isolated brains as well [58]. As anticipated, although both full-body and ex vivo organs showed significantly increased fluorescence in the PPA diet group, the overall average radiant efficiency of ex vivo brains was higher than that of the whole body. We further confirmed *GFAP* gene overexpression and corresponding proteins in the brains of PPA diet progeny versus the control in both the 1 M and 5 M timelines. Concomitantly, under the PPA diet, the neuronal marker Tubulin IIIβ showed a slight decrease in the 1 M study and a significant decrease in the 5 M study. Altogether, the GFAP to Tubulin IIIβ ratio (in both the 1 M and 5 M study) was significantly larger under the PPA diet compared to progeny maintained on a regular chow diet. These findings confirm that a PPA-rich diet triggers astrocyte (the glia subtype responsible for GFAP release) over-proliferation (or possible GFAP overexpression), with an even more pronounced difference at 5 M. The observed differences between 1 M and 5 M could be attributed to prolonged exposure to PPA, which mimics the state of constant dysbiosis reported in ASD patients [59]. Indeed, it was found that antibiotic usage in ASD children may temporarily improve their symptoms, possibly due to the depletion of PPA-producing bacteria in their gut [60,61]. This post-natal gradual exacerbation in the glial phenotype in the PPA group could also be linked to the activation of pre-existing glial cells rather than the de novo formation of additional astrocytes. In fact, MacFabe et al. described the emergence of autistic behavior, reactive astrogliosis, and neuroinflammation in rats treated intracerebroventricularly with PPA [55]. Our study not only corroborates earlier findings but also further establishes the role that PPA (found in the diet or as a byproduct of microbial metabolism) plays in glial proliferation and activation as early as 1 M post-partum.

Our previous in vitro study established that the effect of PPA on hNSC proliferation and differentiation was mediated by the GPR41 receptor, as the inhibition of GPR41 with β-hydroxybutyrate reverted PPA’s effects in vitro [4]. Similarly, this current study shows a significant increase in the gene and protein levels for GPR41 in the 1 M and 5 M timelines. This correlation between increased co-expression of the glial marker and GPR41 in the brain of progeny on a PPA diet corroborates our earlier findings that GPR41 expressed on glial cells allowed for PPA to bind and activate downstream cell proliferation pathways during earlier stages of neural patterning in the mother’s womb.

PTEN/Akt is an important intracellular tandem pathway regulating multiple biological processes, including cell survival, growth, apoptosis, and cancer. Furthermore, PTEN is known to play a critical role in typical brain development, and diminished PTEN levels lead to perturbation in neural patterning and ASD-like symptoms in mice [53,62].

Phosphorylated Akt activates a pro-survival cascade in many cell types, but it is strictly controlled by the PTEN (Phosphatase and tensin homolog) molecule, which inhibits its action by preventing Akt phosphorylation and, therefore, controlling self-renewal [63]. Similar to our earlier in vitro report, a PPA diet appears to reproduce a similar pattern in vivo. In fact, PTEN was significantly decreased in the 5 M study, while phosphorylated Akt was increased (without a change in the total expression of *Akt* RNA). Surprisingly, the 1 M study yielded no significant decrease in PTEN expression and protein concentration nor a change in Akt phosphorylation in the PPA diet progeny versus the control. This could be attributed to the fact that the PTEN/Akt pathway is present in most cell types, and brain tissue is more heterogenous than isolated cells grown on a petri dish; therefore, this provides a less exclusive picture of their expression and more background noise from unrelated neighboring cells. However, at a later time point (5 M) and with subsequent exposure to a PPA diet, a clear shift towards PTEN inhibition and increased Akt phosphorylation is established.

Numerous studies over the last couple of decades have documented encephalitis and neuroinflammation among individuals diagnosed with psychiatric diseases, dementia, and ASD [35,36,38]. In the latter, the state of gliosis and activated microglia (brain immune cells) claim most of the inflammation reported. This current study further confirms that increased glial proliferation under a PPA-rich diet is linked to an increased pro-inflammatory profile. In fact, progeny from dams kept on a PPA diet returned a significant increase in pro-inflammatory *TNF-α* and *IL-6* cytokines and a decrease in anti-inflammatory IL-10 cytokines in their brain extracts, which further confirms our previously published in vitro data [4] as well as extensive literature reports pointing to neuro-inflammation as a contributing factor to ASD development.

The factors contributing to the development of ASD are multifaceted, which adds layers of complexity to the studies aimed at solving the etiology of this puzzling disorder and creates some limitations. To establish a proof of concept, food containing 5000 ppm of PPA was provided ad libitum to mice to maximize the potential effect of PPA on progeny. However, it is well-understood that such a high concentration of PPA on a daily basis is hard to achieve for humans and does not reasonably reflect human eating behavior, which contains varying degrees of unprocessed food; however, it helps mimic an increased amount of PPA being produced by bacteria in gut dysbiosis. For the purpose of this study, the progeny were kept under the same diet as their mothers in order to limit variability and potential confounders. Indeed, ASD children were shown to exhibit high similarity in their microbiota population in comparison to their mothers (75% similarity in vaginally born children and 40% in those born via C-section), potentially due to microbiota transfer from their mothers [64]. Thus, with a continuation of the same diet post-weaning, we aimed to mimic the observed phenomenon, in which increased intestinal PPA in children could be caused by an increase in PPA-producing microorganisms. Additionally, this study does not provide an answer if post-natal exposure to PPA alone could evoke the symptoms presented in this study. Thus, an investigation of the potential difference between pre-natal and post-natal exposure to PPA should be envisioned, in which PPA would be administered to offspring from dams on a control diet post-weaning; however, it is beyond the scope of this current report. Before the isolated brain samples were homogenized, they were carefully and thoroughly washed in PBS to remove blood contamination from circulation that may have accumulated during brain isolation. However, there was a chance, although marginal, that cytokines from systemic circulation may have escaped the blood–brain barrier and contributed to the overall cytokine count obtained. Additionally, it would be of utmost interest to investigate how a maternal PPA-rich diet influences, if at all, the progeny’s microbiota composition and a potential link to gastrointestinal comorbidities found in ASD, adding an additional layer to this already complex endeavor.

In conclusion, this study provides proof of concept that a maternal diet rich in PPA triggers gliosis and neuro-inflammation (as seen in the 1 M study, where the progeny was not directly exposed to PPA-containing chow), which could be subsequently exacerbated by post-weaning exposure of progeny to a PPA-rich diet. These findings are similar to what has been reported in ASD patients, therefore opening avenues for potential preventive measures and ideally offering treatment options to those already affected by this lifelong condition.

## 4. Materials and Methods

### 4.1. Animals and Diet Establishment

All animal work was conducted following the University of Central Florida Institutional Animal Care and Use Committee (UCF-IACUC) guidelines (Animal Use Approval #: PROTO202000002). Transgenic mice overexpressing green fluorescent protein (GFP) under the glia-specific GFAP (Glia fibrillary acid protein) promoter were acquired from Jackson Laboratories (FVB/N-Tg(GFAPGFP)14Mes/J), JAX stock #003257, The Jackson Laboratory, Bar Harbor, ME, USA [65]). Mice expressing the GFAP-GFP construct were used in this study as they allow for the detection of changes in glial proliferation based on GFP signaling, which increases with an increased presence of GFAP both in vitro in the whole animal body and ex vivo in the brain (a detailed explanation is located in Section 4.3). Four weeks prior to mating, through pregnancy and through nursing time, mice were exposed ad libitum to either a control diet (Modified Open Standard Diet With 16 kcal% Fat, Cat# D12020102, Research Diets, Inc. New Brunswick, NJ, USA) or a PPA-rich diet (Modified Open Standard Diet With 16 kcal% Fat with PPA added at a concentration of 5000 mg/kg of total food, Cat# D19071504, custom made by Research Diet Inc., New Brunswick, NJ, USA). The addition of 5000 mg of PPA per kilogram of total food was used to mimic the maximum PPA concentration used in the food industry. Propionic acid (E 280), sodium propionate (E 281), potassium propionate (E 282), and calcium propionate (E 283) are authorized food additives in Europe and the USA at maximal permitted levels (MPLs) ranging from 1000 to 3000 mg/kg in foods [25]. The exact diet composition is included in Table 1 (located below).

Four total groups of offspring were used (with an equal distribution of male and female mice in each group). Out of the four groups, two groups (control and PPA) were sacrificed at 1 month post-partum (designated as 1 M), and two groups were sacrificed at 5 months post-partum (5 M). Mice were humanely sacrificed using 5% Isoflurane (Cat# 792632-250 MG, Sigma-Aldrich, St. Louis, MO, USA) delivered via a nose cone for 10 min followed by cervical dislocation.

The mice designated as 1 M were sacrificed at 4 weeks post-partum and had a body weight between 18 and 22 g. The brain was collected, washed in 1x PBS, and preserved in RNA*later* (Cat# AM7021, Thermo Fisher Scientific, Waltham, MA, USA) at −20 °C for RNA extraction and RT-qPCR procedures (described in detail in Section 4.4). The frozen brain samples were also preserved for protein concentration analysis (described in detail in Section 4.4).

After weaning, the mice designated as 5 M were kept on the same type of diet as their mothers (control or PPA). At 5 months (20 weeks post-partum, weight between 22 and 28 g), they were subjected to whole-body fluorescence imaging (from the GFAP-GFP construct), which is described in detail in Section 4.3. After the mice were humanely sacrificed, the brain was imaged ex vivo using an In Vivo Imaging System (IVIS). Similarly to the 1 M study, the brain was preserved for downstream gene expression and protein level analysis.

### 4.2. Genotyping Using Touchdown PCR to Detect the GFAP/GFP Construct

Progeny from both the control and PPA groups were genotyped to assure the presence of the GFAP-GFP construct via the touchdown PCR protocol using DNA isolated from tail snips. The tail snips were treated with lysis buffer (0.4 mg/mL proteinase K [V3021, Promega, Madison, WI, USA] 10 mM Tris pH 8.00, 100 mM NaCl, 10 mM Ethylenediaminetetraacetic acid [EDTA], 0.5% Sodium Dodecyl Sulfate [SDS]) overnight in a 56 °C water bath. Then, DNA was precipitated with saturated NaCl (the sample was mixed with half of the volume of a 6 M NaCl solution), centrifugated, and washed with 70% ethanol. The remaining DNA pellet was air dried and resuspended in TE buffer (10 mM Tris-HCl pH 8.0, 0.1 mM EDTA, Cat# 12090015, Thermo Fisher Scientific, Waltham, MA, USA). An amount of 200 ng of DNA was used for each reaction, in conjunction with the following primers: Forward: 5′ ACT CCT TCA TAA AGC CCT CG 3′ and Reverse: 5′ AAG TCG ATG CCC TTC AGC TC 3′. PCR was performed using a PCR master mix (*Taq*, Cat# M7502, Promega, Madison, WI, USA) in a thermocycler (Mastercycler Gradient, Eppendorf, Hamburg, Germany) using the following touchdown settings: 95 °C for 6 min, 65 °C for 1.5 min, and 68 °C for 1.5 min. The cycle was then repeated with the annealing temperature decreased by 0.5 °C per cycle until it reached 60 °C, after which another set of cycles (94 °C for 1 min, 65 °C for 1.5 min, and 72 °C for 1.5 min) was repeated 25 times. The PCR product was subjected to 50 min electrophoresis on 1.5% agarose gel pre-mixed with loading dye (Cat# R0611 Thermo Fisher Scientific, Waltham, MA, USA) and ethidium bromide (Cat# H504, Promega, Madison, WI, USA). A FastRuler Low Range DNA Ladder (SM1103, Thermo Fisher Scientific, Waltham, MA, USA) was used to determine the PCR product length. The expected PCR product length was 498 bp. DNA extracted from male breeders and female breeders (purchased from Jackson laboratories) was used as positive and negative controls, respectively. Only progeny positive for GFAP-GFP were tested with live fluorescence imaging (IVIS), in which the average radiant efficiency of the whole body and ex vivo brains was measured.

### 4.3. In Vivo and Ex Vivo IVIS Imaging

Mice positive for the GFAP-GFP construct (determined by the Touchdown PCR technique) were selected for whole-body imaging and ex vivo brain fluorescence imaging using the In Vivo Imaging System (IVIS, Lumina S5, Perkin Elmer, Waltham, MA, USA) connected to an isoflurane delivery system. Animals were sedated throughout the procedure. Fluorescent imaging was also obtained from the ex vivo brain isolated from the humanely sacrificed mice at 5 months (as described in Section 4.1). Prior to imaging, the brains were 1X PBS-washed and moisture-dampened. They were not dipped in media to avoid any media fluorescence interference. An empty well was also imaged to demonstrate an absence of fluorescence from the walls of the wells. The brains were positioned with the hemispheres facing upwards and the cerebellum and brain stem facing downwards (which were included). The intensity was adjusted to remain constant throughout the experiment and across groups, and the average radiant efficiency was obtained (Living Image software, version 4.7.0, Perkin Elmer, Waltham, MA, USA).

### 4.4. Gene Expression

Tissue (brain) intended for RNA extraction was preserved with RNA*later* and kept at −20 °C until processed. Tissues were minced with a scalpel blade and homogenized with a mechanical tissue grinder (Cat# 02-542-10, Hampton, NH, USA) and submerged in TRI Reagent™ Solution (Cat# AM9738, Ambion, Austin, TX, USA). After the addition of chloroform (Serva, Heidelberg, Germany) and centrifugation, the aqueous layer containing RNA was isolated and the total RNA was subsequently precipitated with ice-cold 100% isopropyl alcohol and centrifugation (30 min, 10,000 RCF, 4 °C). The supernatant was discarded, and the pellet was washed twice with 70% ethyl alcohol and air-dried. The RNA pellets were resuspended in diethylpyrocarbonate (DEPC)-treated nuclease-free water, and the concentration was measured with NanoDrop One^C^ (Thermo Fisher Scientific, Waltham, MA, USA). cDNA was synthesized using 1 μg of pure RNA in a 20 μL reaction. A 2× High-Capacity cDNA Reverse Transcription Kit (Cat# 4368814, Applied Biosystems, Waltham, MA, USA) was used to synthesize cDNA following the standard manufacturer’s protocol. For qPCR, cDNA was diluted twenty-fold with nuclease-free water. The RT-PCR reaction solution was assembled on ice to contain 2 × PowerUp™ SYBR™ Green Master Mix for qPCR (Cat# A25741, Applied Biosystems, Waltham, MA, USA) and primers purchased from Bio-Rad Laboratories (Hercules, CA, USA) that have a proprietary sequence (T*UBB3*: qMmuCID0018119, *GFAP*: qMmuCID0020163, *IL-6*: qMmuCED0045760, *IL-10*: qMmuCID0015452, *Tnf-α*: qMmuCED0004141, *GPR41*: qMmuCED0003601, *Akt1*: qMmuCED0044805, PTEN: qMmuCID0005543). The reaction was carried out in a 96-well plate in the StepOne Plus Real-Time PCR System (Applied Biosystems, Waltham, MA, USA). Gene expression was normalized to the *GAPDH* housekeeping gene (Bio-Rad GAPDH: qMmuCED0027497). Each sample was run in duplicates or triplicates. Relative gene expression was assessed by using the equation 2^(−∆∆CT)^, in which ∆CT = CT_target gene_ − CT_reference gene(GAPDH)_, and ∆∆CT = ∆CT_control_ − ∆CT_treatment_. The data are presented as fold change [66].

### 4.5. Protein Levels

Frozen brain tissues were minced with a scalpel blade and submerged in a cell lysis buffer supplied with proteases and phosphatases inhibitors (Cat# 9803, Cell Signaling Technology, Danvers, MA, USA) and homogenized with a mechanical tissue grinder. Following homogenization, the samples were subjected to sonification to assure lysis of all cells (5 s, 50% amplitude, 450 Sonifier Analog Cell Disruptor, Branson, Brookfield, CT, USA). The samples were centrifugated (10 min, 10,000 RCF, 4 °C) to remove debris, and total protein concentration was measured using a Bradford assay and a bovine serum albumin standard curve (Quick Start™ Bradford Protein Assay Kit, Cat# 5000201, Bio-Rad, Hercules, CA, USA). The normalized amount of protein (the same for each sample) was used for the enzyme-linked immunosorbent assay (ELISA). ELISAs were performed in accordance with the included manufacturer’s instructions. The following commercially available kits were used: GFAP: Cat# LS-F23063, Lifespan Biosciences (LS-Bio), Lynnwood, WA, USA; Tubulin-IIIβ: Cat# LS-F18199-1, LS-Bio; GPR41: Cat# LS-F9213-1, LS-Bio; PTEN: LS-Bio Cat# LS-F8566-1; IL-10: Cat# ab255729, Abcam, Cambridge, UK; p-Akt: PathScan^®^ Phospho-Akt (Thr308), Cat# 7252, Cell signaling Technology, Danvers, MA, USA.

### 4.6. Statistical Analysis

All statistical analyses were performed using GraphPad Prism 10.1.0 software (GraphPad Software, Boston, MA, USA). Prior to performing statistical analysis, all data were tested for normal distribution using the Shapiro–Wilk normality test. The significance was determined using an unpaired two-tailed *t*-test (for normally distributed data) and the Mann–Whitney U test (for non-normally distributed data). A *p*-value lower than 0.05 (*), 0.01 (**), 0.001 (***), or 0.0001 (****) was considered significant.

## Figures and Tables

**Table 1:** A description of the diet provided to animals in either the control or PPA groups, including the detailed composition and caloric values of each component. The data were provided by the chow manufacturer (Research Diets, Inc. New Brunswick, NJ, USA).

Product #	Control (Cat# D12020102)		PPA (Cat# D19071504)	
Research Diets, Inc.				
	gm%	kcal%	gm%	kcal%
Protein	19.9	20	19.8	20
Carbohydrate	63.8	64	63.4	64
Fat	6.9	16	6.8	26
Total		100		100
kcal/gm	3.96		3.94	
Ingredient	gm	kcal	gm	kcal
Casein	200	800	200	8000
L-Cystine	3	12		
Corn Starch	346	1384	346	1384
Maltodextrin 10	45	180	45	180
Dextrose	250	1000	250	1000
Cellulose, BW200	50	0	50	0
Soybean Oil	70	630	70	630
Mineral Mix S10026	10	0	10	0
Dicalcium Phosphate	13	0	13	0
Calcium Carbonate	5.5	0	5.5	0
Potassium Citrate, 1 H_2_O	16.5	0	16.5	0
Vitamin Mix V10001	10	40	10	40
Choline Bitartrate	2	0	2	0
Sodium Propionate	0	0	5.14	0
Yellow Dye #5, FD&C	0.05	0	0.025	0
Blue Dye #1, FD&C		0	0.025	0
Total	1021.05	4046	1026.19	4046

## Data Availability

Raw data are available upon request.
